# Demography and commonly recorded clinical conditions of Chihuahuas under primary veterinary care in the UK in 2016

**DOI:** 10.1186/s12917-020-2258-1

**Published:** 2020-02-12

**Authors:** Dan G. O’Neill, Rowena M. A. Packer, Meghan Lobb, David B. Church, Dave C. Brodbelt, Camilla Pegram

**Affiliations:** 1 Pathobiology and Population Science, The Royal Veterinary College, Hawkshead Lane, North Mymms, Hatfield, Herts AL9 7TA UK; 2 Clinical Science and Services, The Royal Veterinary College, Hawkshead Lane, North Mymms, Hatfield, Herts AL9 7TA UK

**Keywords:** VetCompass, Electronic patient record, EPR, Breed, Dog, Epidemiology, Primary-care, Veterinary, Pedigree, Purebred

## Abstract

**Background:**

The Chihuahua, the world’s smallest dog breed, is a popular breed in the UK today. The VetCompass™ Programme collates de-identified clinical records from primary-care veterinary practices in the UK for epidemiological research. This study aimed to characterise the demography, age at death and common disorders of Chihuahuas under primary veterinary care during 2016 in the UK.

**Results:**

Chihuahuas comprised 11,647/336,865 (3.46%) dogs under veterinary care during 2016. The annual proportional birth rate for Chihuahuas rose from 1.01% in 2005 to 5.35% in 2016. Median adult bodyweight was 3.4 kg (interquartile range [IQR]: 2.7–4.3, range 0.8–9.8).).

Median age was 2.8 years (interquartile range [IQR] 1.6–4.9). Median age at death from this young expanding population was 8.2 years (IQR 3.5–12.1). Females (10.2 years) outlived males (6.9 years) (Mann-Whitney U test: *P* = 0.005). The most common grouped causes of death were heart disease (18.8%, 95% CI: 10.9–29.0), lower respiratory tract disorder (16.3%, 95% CI: 8.9–26.2) and traumatic injury (13.8%, 95% CI: 7.1–23.3). The most common specific disorders were periodontal disease (13.5%, 95% CI: 12.6–14.4), obesity (5.9%, 95% CI: 5.3–6.5), retained deciduous dentition (5.7%, 95% CI: 5.1–6.4), anal sac impaction (4.9%, 95% CI: 4.4–5.5) and aggression (4.2%, 95% CI: 3.7–4.8). Among the 28 most common fine-level disorders, males had statistically (*P* <  0.005) higher probability than females for 5 disorders (aggression, heart murmur, otitis externa, conjunctivitis and upper respiratory tract infection). There were no disorders with statistically (*P* <  0.005) higher prevalence in females.

**Conclusions:**

This study documented rising ownership and a currently youthful population of Chihuahuas in the UK. These results suggest that the Chihuahua is currently undergoing a popularity boom but veterinarians need to be watchful for welfare issues related to impulse purchase of Chihuahua puppies by owners with limited experience of dog care. Periodontal disease, obesity, retained deciduous dentition, anal sac impaction and aggression were identified as common health issues within the breed. The unique veterinary care needs of this popular miniature breed suggest that veterinarians should consider the value of advanced training in anesthesia and dentistry in small-sized dogs.

## Background

The Chihuahua, the world’s smallest dog breed, takes its name from the Mexican state where the breed became fashionable in the late nineteenth Century. The breed offers two varieties, the smooth coat and the long coat, that are considered distinct variants by the UK Kennel Club but share otherwise similar physical attributes [1]. In the UK, the Chihuahua overall was the 16th most commonly microchipped breed between 2004 and 2014 [2]. Kennel Club breed registration data identified a gradual rise in Chihuahua (smooth coat and long coat considered together) registration numbers in the UK from 1955 until the mid-1970s. From 1973, breed registration numbers fluctuated with a gradual decrease over the following thirty years before sharply rising from 2003 onwards [3].

The UK Kennel Club breed standard describes the Chihuahua as ‘small, dainty and compact’ with a bodyweight of up to 2.7 kg [1]. The Kennel Club reports the Chihuahua lifespan as spanning over 12 years [1]. Analysis of UK primary-care veterinary records contrastingly reported a median age at death of 7.1 years derived from the ages at death recorded in the clinical records but this low age at death may be biased downwards by a relatively youthful UK population [4]. A UK pedigree dog breed health survey in 2014 included data on 131 smooth coat Chihuahuas and 124 long coat Chihuahuas and reported youthful median ages for smooth coat Chihuahuas at 2 years and for long coat Chihuahuas at 3 years that suggested the breed was growing in popularity [5, 6].

Prevalence is an absolute value that defines the overall frequency of a condition whereas predisposition is a relative value that describes the risk in one group in comparison to another [7]. A textbook that reviewed the general literature identified that the Chihuahua has reported predispositions to 21 disorders including degenerative mitral valve disease, patellar luxation, hydrocephalus, corneal ulceration, dystocia and tracheal collapse, although these studies varied widely in study design, date, geographical location and comparator groups [8]. However, data on disorder prevalence within Chihuahuas is relatively limited. The most commonly reported disorders in smooth coat Chihuahuas were patellar luxation, trachea disorder, food allergy and regular reverse sneezing [5], whilst cryptorchidism, regular reverse sneezing, anal gland infection, haemorrhagic gastroenteritis and patellar luxation were most common in long coat Chihuahuas [6]. The Kennel Club has reported the Chihuahua as a Breed Watch category 2 breed, with incorrect dentition noted as a point of concern for special attention by judges [9].

The discovery and reporting of sex-based associations with disease and longevity can highlight opportunities for targeted focus on preventive and remedial control within sexes to optimise health and welfare improvements [10]. It is also important to consider the age structure of study populations to ensure safe interpretation of disorder and age at death results between breeds that may have widely different popularity trends [11]. Some previous breed-specific studies have reported on effects associated with age and sex in Greyhounds and Miniature Schnauzers [12, 13]. However, to date, there has been limited reporting of health differences associated with age and sex for Chihuahuas.

This study aimed to report the demography, age at death and common disorders of Chihuahuas under primary veterinary care in the UK based on anonymised veterinary clinical data derived from the VetCompass™ Programme [14]. Health effects associated with age and sex were of special interest. These findings can be applied by owners and veterinary practitioners to predict health and welfare opportunities for Chihuahuas.

## Results

### Demography and mortality

**Fig. 1:**
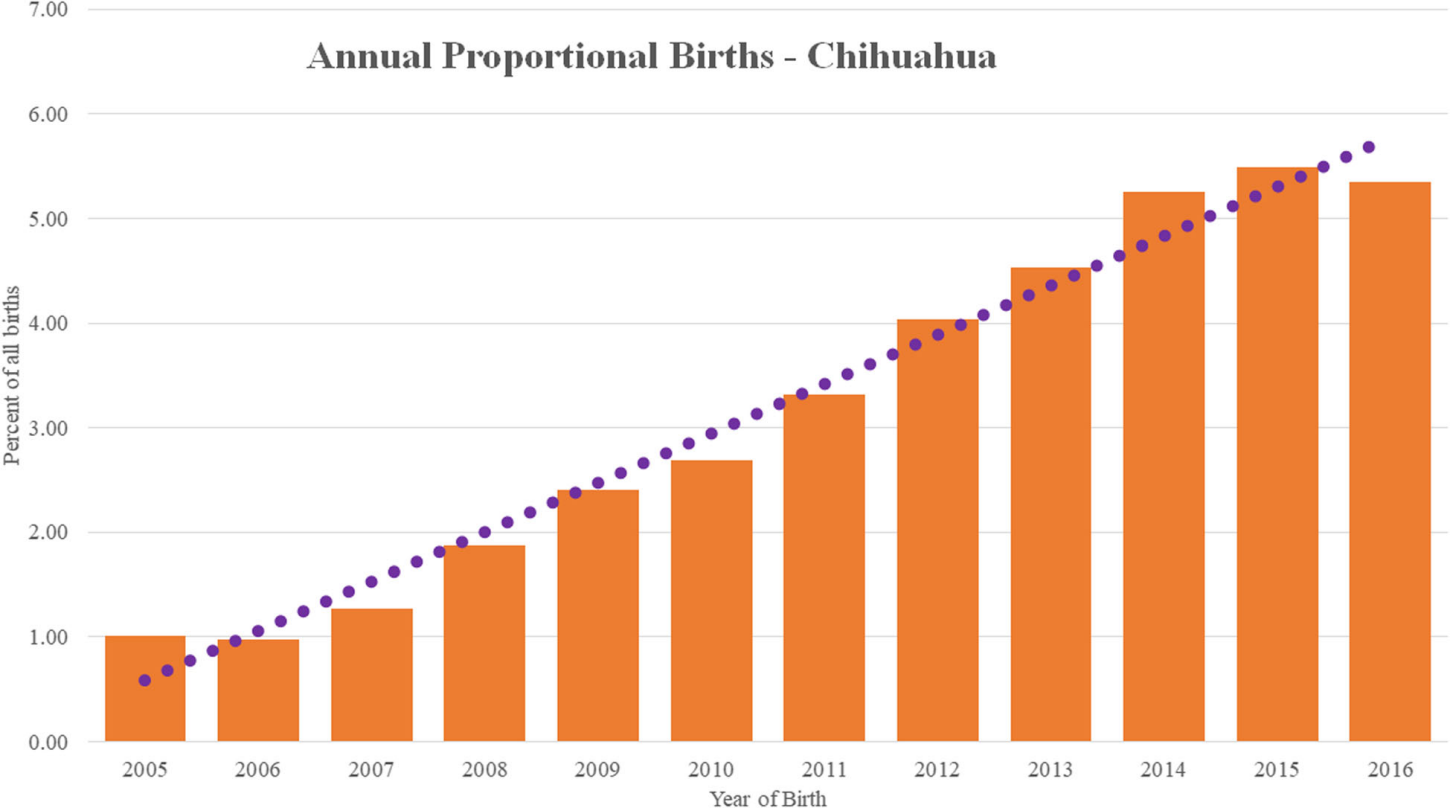
Annual proportional birth rates (2005–2016) for Chihuahuas (*n* = 11,647) among all dogs (*n* = 336,865) under UK primary veterinary care from January 1st 2016 to December 31st, 2016 at practices participating in the VetCompass™ Programme. The image is overlaid with a linear trend line

The study population of 336,865 dogs under veterinary care during 2016 included 11,647 (3.46%) Chihuahuas attending 438 clinics in the VetCompass database. Of these Chihuahuas with information available, 5780 (49.8%) were female and 3127 (26.9%) across both sexes were neutered. Annual proportional birth rates showed that Chihuahuas increased steeply from 1.01% of the annual VetCompass birth cohort in 2005 to 5.35% in 2016 (Fig. 1). Males were significantly more likely to be neutered than females (31.8% versus 22.0%, chi-square test: *P* <  0.001). The median age of the Chihuahuas overall was 2.8 years (interquartile range [IQR] 1.6–4.9, range 0.1–18.6). There were 6388 (65.2%) dogs recorded with a single colour, 3261 (33.3%) recorded with 2 colours, 140 (1.4%) with 3 colours and 4 (0.04%) with four colours. Of the dogs with a single colour, the most common colours were fawn/cream (*n* = 3716, 58.2%), chocolate (717, 11.2%) and white (649, 10.2%).

**Fig. 2:**
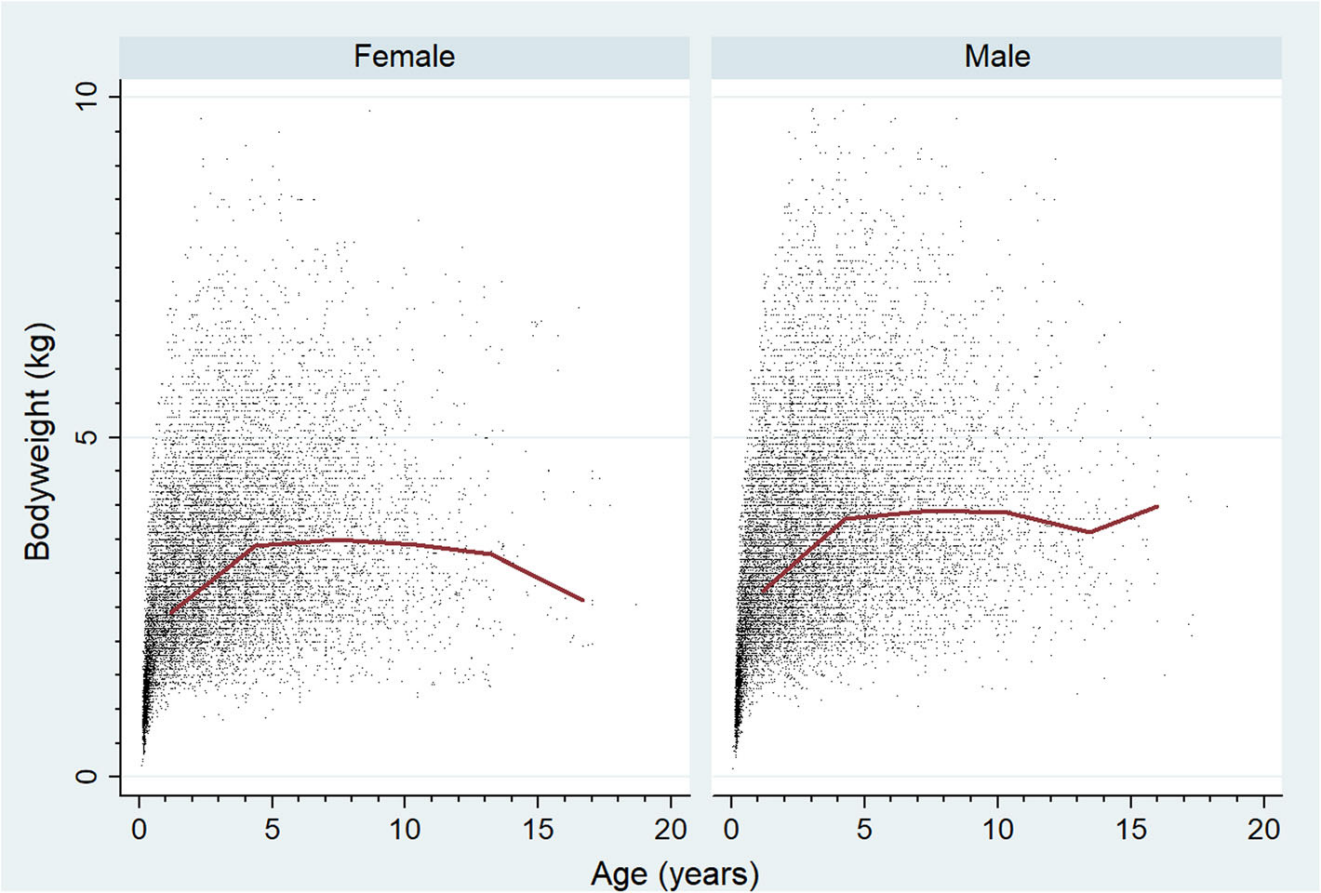
Bodyweight growth curves overlaid with a cross medians line plot for female and male Chihuahuas under UK primary veterinary care from January 1st 2016 to December 31st, 2016 at practices participating in the VetCompass™ Programme (14,531 bodyweight values from 4522 females and 16,259 bodyweight values from 4770 males)

The median adult bodyweight overall was 3.4 kg (IQR: 2.7–4.3, range 0.8–9.8). The median adult bodyweight of males (3.7 kg, IQR: 2.9–4.6, range 1.1–9.8) was heavier than females (3.2 kg, IQR: 2.6–4.0, range 0.8–9.8) (Mann-Whitney U test: *P* <  0.001) (Table 1). The median bodyweight across all ages for males (3.2 kg, IQR: 2.3–4.2, range: 0.1–9.9) was higher than for females (2.8 kg, IQR: 2.0–3.8, range: 0.2–9.8) (Mann-Whitney U test: *P* <  0.001). Bodyweight growth curves based on 14,531 bodyweight values from 4522 females and 16,259 bodyweight values from 4770 males showed that Chihuahua puppies grow rapidly during their first year but continue to gain further weight up to 4 years of age (Fig. 2). The proportional completeness for each variable was: colour 84.1%, sex 99.7%, neuter 99.7%, age 97.4% and bodyweight at any age 79.9%.

**Table 1:** Demography of 11,647 Chihuahuas under primary veterinary care at practices participating in the VetCompass™ Programme in the UK from January 1st to December 31st, 2016. *Count covers dogs with available data

Variable	Category	Count*	Percent
Sex	Female	5780	49.8
	Male	5831	50.2
Female neuter	Entire	4507	78.0
	Neutered	1273	22.0
Male neuter	Entire	3977	68.2
	Neutered	1854	31.8
Female adult bodyweight (aged ≥18 months) (kg)	< 2.0	251	7.5
	2.0 to < 3.0	1113	33.3
	3.0 to < 4.0	1109	33.1
	4.0 to < 5.0	531	15.9
	5.0 to < 6.0	227	6.8
	≥ 6.0	116	3.5
Male adult bodyweight (aged ≥18 months) (kg)	< 2.0	128	3.7
	2.0 to < 3.0	835	24.0
	3.0 to < 4.0	1111	31.9
	4.0 to < 5.0	772	22.2
	5.0 to < 6.0	378	10.9
	≥ 6.0	255	7.3
Age (years)	< 1.0	545	4.8
	1.0 to < 2.0	3338	29.4
	2.0 to < 3.0	2083	18.4
	3.0 to < 5.0	2662	23.5
	5.0 to < 7.0	1391	12.3
	7.0 to < 9.0	763	6.7
	9.0 to < 11.0	309	2.7
	≥ 11.0	252	2.2
Colour (for dogs recorded with a single colour)	Fawn/cream	3716	58.2
	Chocolate	717	11.2
	White	649	10.2
	Black	398	6.2
	Blue	231	3.6
	Red	222	3.5
	Gold	199	3.1
	Sable	146	2.3
	Brindle	62	1.0
	Silver	30	0.5
	Merle	18	0.3

There were 102 deaths recorded during the study. The median age at death overall from this young expanding population of Chihuahuas was 8.2 years (IQR 3.5–12.1, range 0.1–17.0). The median age at death of females (10.2 years, IQR 7.2–12.7, range 1.9–17.0, *n* = 37) was greater than males (6.9 years, IQR 1.4–10.6, range 0.1–17.0, *n* = 56) (Mann-Whitney U test: *P* = 0.005). From 91 (89.2%) deaths that showed the method of death, there were 62 (68.1%) deaths by euthanasia and 29 (31.9%) unassisted deaths. The cause of death was recorded for 80 (78.4%) deaths. The most common grouped-precision causes of death were heart disease (*n* = 15, prevalence 18.8%), lower respiratory tract disorder (13, 16.3%) and traumatic injury (11, 13.8%) (Table 2).

**Table 2:** Mortality in Chihuahuas with a recorded cause of death under primary veterinary care at UK practices participating in the VetCompass™ Programme from January 1st to December 31st, 2016 (*n* = 80)

Grouped-level disorder	Count	Percent	95% CI
Heart disease	15	18.8	10.9–29.0
Lower respiratory tract disorder	13	16.3	8.9–26.2
Traumatic injury	11	13.8	7.1–23.3
Brain disorder	9	11.3	5.3–20.3
Enteropathy	5	6.3	2.1–14.0
Poor quality of life	4	5.0	1.4–12.3
Renal disease	3	3.8	0.8–10.6
Behaviour disorder	2	2.5	0.3–8.7
Endocrine disorder	2	2.5	0.3–8.7
Mass-associated disorder	2	2.5	0.3–8.7
Urinary system disorder	2	2.5	0.3–8.7
Abdominal disorder	1	1.3	0.0–6.8
Adverse reaction to drug	1	1.3	0.0–6.8
Collapsed	1	1.3	0.0–6.8
Complication associated with clinical care procedure	1	1.3	0.0–6.8
Haematopoietic disorder	1	1.3	0.0–6.8
Hepatopathy	1	1.3	0.0–6.8
Lethargy	1	1.3	0.0–6.8
Neoplasia	1	1.3	0.0–6.8
Pancreatic disorder	1	1.3	0.0–6.8
Parasite infestation	1	1.3	0.0–6.8
Skin disorder	1	1.3	0.0–6.8
Spinal cord disorder	1	1.3	0.0–6.8

### Disorder prevalence

The EPRs of a random sample of 5660/11,647 (48.6%) Chihuahuas were manually examined to extract all recorded disorder data for 2016. At least one disorder was recorded during 2016 for 3112 (55.0%) Chihuahuas. The other 45.0% did not have any disorder recorded and may have received only prophylactic or no direct veterinary care during 2016. The median annual disorder count per Chihuahua during 2016 was 1 disorder (IQR 0–1, range 0–10). The median annual disorder count was higher in males (1, IQR 0–2, range 0–10) than in females (1, IQR 0–1, range 0–8) (Mann-Whitney U test: *P* <  0.001).

There were 5333 unique disorder events recorded during 2016 spanning 352 separate fine-level disorder terms. The most prevalent fine-level precision disorders recorded were periodontal disease (*n* = 762, prevalence 13.5%, 95% CI: 12.6–14.4), obesity (333, 5.9%, 95% CI: 5.3–6.5), retained deciduous dentition (324, 5.7%, 95% CI: 5.1–6.4), anal sac impaction (280, 4.9%, 95% CI 4.4–5.5) and aggression (238, 4.2%, 95% CI 3.7–4.8). Among the 28 most common fine-level disorders, males had statistically (chi-square test, *P* <  0.05) higher probability than females for 5 disorders (aggression, heart murmur, otitis externa, conjunctivitis and upper respiratory tract infection). There were no disorders with higher prevalence in females. The median age of dogs recorded with each of the 28 most common fine-level disorders varied from 1.5 years for alopecia to 8.9 years for heart murmur (Table 3).

**Table 3:** Prevalence of the most common disorders at a *fine-level of diagnostic precision* recorded in Chihuahuas (*n* = 5660) under primary veterinary care at UK practices participating in the VetCompass™ Programme from January 1st to December 31st, 2016. The *P*-value reflects prevalence comparison between females and males. *CI confidence interval

Fine-level disorder	Count	Overall prevalence %	95% CI*	Female prevalence %	Male prevalence %	*P*-Value	Median age (years)
Periodontal disease	762	13.5	12.6–14.4	13.3	13.7	0.650	5.2
Obesity	333	5.9	5.3–6.5	6.3	5.6	0.264	4.4
Retained deciduous dentition	324	5.7	5.1–6.4	5.4	6.1	0.269	1.6
Anal sac impaction	280	4.9	4.4–5.5	4.9	5.1	0.728	3.8
Aggression	238	4.2	3.7–4.8	3.5	4.9	0.008	3.5
Patellar luxation	224	4.0	3.5–4.5	4.2	3.7	0.345	2.9
Cryptorchidism (males only)	110	3.9	3.2–4.6	~	~	~	1.6
Overgrown nail(s)	187	3.3	2.9–3.8	3.6	3.1	0.279	3.7
Flea infestation	162	2.9	2.4–3.3	2.8	3.0	0.596	2.9
Heart murmur	115	2.0	1.7–2.4	1.3	2.8	< 0.001	8.9
Anal sac infection	100	1.8	1.4–2.1	1.4	2.1	0.053	4.1
Undesirable behaviour	83	1.5	1.2–1.8	1.4	1.6	0.485	2.5
Diarrhoea	76	1.3	1.1–1.7	1.1	1.5	0.189	2.2
Otitis externa	74	1.3	1.0–1.6	0.9	1.7	0.012	4.0
Lameness	63	1.1	0.9–1.4	1.0	1.2	0.571	2.5
Gastroenteritis	61	1.1	0.8–1.4	1.1	1.1	0.949	1.9
Vomiting	61	1.1	0.8–1.4	1.1	1.1	0.949	2.1
Dystocia (females only)	31	1.1	0.8–1.6	~	~	~	2.8
Pruritus	46	0.8	0.6–1.1	0.8	0.8	0.955	3.0
Corneal ulceration	46	0.8	0.6–1.1	0.9	0.7	0.516	3.4
Conjunctivitis	43	0.8	0.6–1.0	0.4	1.1	0.004	1.7
Claw injury	39	0.7	0.5–0.9	0.6	0.7	0.667	3.1
Infectious canine tracheobronchitis	36	0.6	0.4–0.9	0.5	0.8	0.105	2.1
Upper respiratory tract infection	36	0.6	0.4–0.9	0.4	0.9	0.009	3.2
Dental disease	35	0.6	0.4–0.9	0.6	0.6	0.904	4.2
Alopecia	33	0.6	0.4–0.9	0.5	0.6	0.634	1.5
Melaena	33	0.6	0.4–0.9	0.5	0.6	0.634	2.7
Spinal pain	31	0.6	0.4–0.8	0.4	0.7	0.115	5.5

Prevalence values were reported for 15 disorders that featured among the 10 most common disorders recorded at a *fine-level of diagnostic precision* within at least one of three age bands: under 2 years, 2–7 years and over 7 years. There were 1918 dogs aged under 2 years, 2988 dogs aged from 2 to 7 years and 664 dogs aged over 7 years. Overall, 11/15 (73.3%) of these disorders showed statistically significant (chi-square or Fisher’s exact test, *P* <  0.05) differences in prevalence between the three age bands (Table 4).

**Table 4:** Prevalence of the combined list from the 10 most common disorders recorded at a *fine-level of diagnostic precision* within each of three age bands (under 2 years, 2–7 years, over 7 years) in Chihuahuas under primary veterinary care at UK practices participating in the VetCompass™ Programme from January 1st to December 31st, 2016. The P-value reflects prevalence comparison between the three age bands using the statistical test described. Under 2 years *N* = 1918, 2–7 years *N* = 2988, over 7 years *N* = 664). *CI confidence interval

Fine-level disorder	< 2 yrs.: Rank	< 2 yrs.: N (%)	2–7 yrs.: Rank	2–7 yrs.: N (%)	> 7 yrs.: Rank	> 7 yrs.: N (%)	*P*-Value	Statistical Test
Aggression	4	55 (2.87)	4	143 (4.79)	5	33 (4.97)	0.002	Chi squared
Anal sac impaction	6	51 (2.66)	3	178 (5.96)	4	50 (7.53)	< 0.001	Chi squared
Anal sac infection	29	10 (0.52)	9	69 (2.31)	8	20 (3.01)	< 0.001	Chi squared
Undesirable behaviour	11	31 (1.62)	10	47 (1.57)	31	5 (0.75)	0.246	Chi squared
Cryptorchidism (males only)	2	76 (7.45)	14	29 (2,00)	42	4 (1.16)	< 0.001	Fisher’s exact
Diarrhoea	7	34 (1.77)	12	37 (1.24)	33	5 (0.75)	0.102	Chi squared
Gastroenteritis	9	33 (1.72)	22	21 (0.7)	20	7 (1.05)	0.004	Chi squared
Chronic cardiac disease	259	0 (0)	288	0 (0)	9	19 (2.86)	< 0.001	Fisher’s exact
Heart murmur	61	5 (0.26)	16	27 (0.9)	2	82 (12.35)	< 0.001	Chi squared
Obesity	8	34 (1.77)	2	238 (7.97)	3	61 (9.19)	< 0.001	Chi squared
Overgrown nail(s)	10	33 (1.72)	6	119 (3.98)	6	30 (4.52)	< 0.001	Chi squared
Flea infestation	5	55 (2.87)	8	86 (2.88)	10	18 (2.71)	0.972	Chi squared
Patellar luxation	3	61 (3.18)	5	133 (4.45)	7	30 (4.52)	0.068	Chi squared
Periodontal disease	12	31 (1.62)	1	498 (16.67)	1	226 (34.04)	< 0.001	Chi squared
Retained deciduous dentition	1	217 (11.31)	7	105 (3.51)	320	0 (0)	< 0.001	Fisher’s exact

There were 55 distinct grouped-level precision disorder terms recorded. The most prevalent grouped-level precision disorders were dental (*n* = 1075, prevalence: 19.0%, 95% CI: 18.0–20.0), behavioural (373, 6.6%, 95% CI: 6.0–7.3), anal sac (363, 6.4%, 95% CI: 5.8–7.1), musculoskeletal (340, 6.0%, 95% CI 5.4–6.7) and obesity (333, 5.9%, 95% CI: 5.3–6.5). Among the 20 most common grouped disorders, males had statistically (chi-square test, *P* <  0.05) higher probability than females for 6 disorders: behavioural, ophthalmologic, upper respiratory tract, cardiac, aural and spinal cord. There were no disorders with higher prevalence in females. The median age of dogs recorded with each of the 20 most common grouped-level disorders varied from 1.6 years for male reproductive to 9.1 years for cardiac (Table 5).

**Table 5:** Prevalence of the most common disorders at a *grouped-level of diagnostic precision* recorded in Chihuahuas (*n* = 5660) under primary veterinary care at UK practices participating in the VetCompass™ Programme from January 1st to December 31st, 2016. The P-value reflects prevalence comparison between females and males. *CI confidence interval

Grouped-level disorder	Count	Overall prevalence %	95% CI*	Female prevalence %	Male prevalence %	*P*-Value	Median age
Dental	1075	19.0	18.0–20.0	18.5	19.5	0.342	4.1
Behavioural	373	6.6	6.0–7.3	5.6	7.6	0.004	3.1
Anal sac	363	6.4	5.8–7.1	6.0	6.9	0.159	3.9
Musculoskeletal	340	6.0	5.4–6.7	6.0	6.1	0.859	3.2
Obesity	333	5.9	5.3–6.5	6.3	5.6	0.264	4.4
Enteropathy	314	5.5	5.0–6.2	5.2	5.9	0.260	2.4
Dermatological	271	4.8	4.2–5.4	4.6	5.0	0.586	3.5
Male reproductive (males only)	120	4.2	3.5–5.0	~	~	~	1.6
Claw/nail	226	4.0	3.5–4.5	4.2	3.8	0.420	3.7
Female reproductive (females only)	91	3.3	2.6–4.0	~	~	~	3.2
Parasitic	190	3.4	2.9–3.9	3.2	3.5	0.534	2.7
Ophthalmologic	175	3.1	2.7–3.6	2.6	3.6	0.034	3.2
Upper respiratory tract	165	2.9	2.5–3.4	2.4	3.4	0.029	3.2
Cardiac	140	2.5	2.1–2.9	1.6	3.4	< 0.001	9.1
Traumatic injury	111	2.0	1.6–2.4	1.7	2.2	0.177	2.2
Aural	82	1.4	1.2–1.8	1.1	1.8	0.018	4.0
Brain	66	1.2	0.9–1.5	0.9	1.4	0.096	5.1
Mass associated	58	1.0	0.8–1.3	1.1	1.0	0.554	5.5
Complication associated with clinical care	49	0.9	0.6–1.1	0.9	0.9	0.932	2.9
Spinal cord	48	0.8	0.6–1.1	0.6	1.1	0.024	4.9

## Discussion

This 2016 period cross-sectional study is the largest study to date to report on Chihuahua health using primary-care veterinary data. The study characterised the demography of 11,647 Chihuahuas and also the age at death and commonly recorded disorders of 5660 Chihuahuas. The results highlight a sharply increasing ownership trend for Chihuahuas in the UK over the past decade, and a youthful current population. The most common grouped causes of mortality were heart disease, lower respiratory tract disorder and traumatic injury. The most prevalent fine-level disorders of Chihuahuas were periodontal disease, obesity, retained deciduous dentition, anal sac impaction and aggression. These results reiterate the power of primary-care records to highlight key issues within breeds and expand the evidence-base within breed-related health in dogs [15].

Annual proportional birth rates identified steeply rising ownership of Chihuahua in the wider population of dogs in the UK, rising five-fold from 1.01% of dogs born in 2005 to 5.35% of dogs born in 2016. The Kennel Club breed registration statistics somewhat reflect the earlier popularity trend identified in the current study, with increasing registrations from 2003 and 2014 but showing a slight decline after this. Kennel Club registrations are estimated to account for only around 30% of the total population of dogs in the UK and therefore may not accurately reflect ownership trends in the wider dog population [3, 16]. Given that the capability of breeders registered with the Kennel Club to increase puppy production rapidly is likely to be limited, this suggests that the bulk of puppies needed during sudden spikes in popularity are likely to come from sources outside the Kennel Club arena. Increased demand for breeds, such as the Chihuahua, with extreme conformational features can be exponentially detrimental to welfare because intrinsic disorder predispositions are compounded by extrinsic welfare issues associated their popularity. Increasing demand for any dog breed can promote suboptimal breeding and welfare standards when national and international breeders and suppliers race to meet the rapidly-rising consumer demand [17, 18]. Recent studies have documented increased incidence of behavioural and emotional problems in dogs born in high-volume commercial breeding establishments [19].

Social influence has a major effect on the popularity of individual dog breeds and is often related to media exposure e.g. breeds that feature in movies [20, 21]. A recent study of American dog breeds, including the Chihuahua, indicated that breed popularity now appears to lack direct associations with functional traits (e.g. health, trainability) but instead displays a concerning tendency for more popular breeds to have greater numbers of inherited disorders and behavior problems [22]. At the individual dog level, such health problems may paradoxically facilitate positive dog-owner relationships. Chihuahua owners are demonstrated to share closer attachments to their dogs than Cairn terrier owners, with higher levels of health and behavior problems positively associated with closer attachment for owners of Chihuahuas [18].

Breeds such as the Chihuahua have been selected to exhibit baby-like features into their adult years; the UK breed standard calls for eyes that are “*large, round, but not protruding; set well apart*” and a head that has a “*well rounded ‘apple dome’ skull*” [23]. Baby-like physical features including large, round, wide-set eyes combined with rounded faces are thought to elicit an unconscious ‘cute-response’ in some people [24]. Such features are associated with young animals and it is believed that humans are innately inclined to care for young animals as they do for children [25].

The median age at death of the Chihuahuas that died in the current study was 8.2 years, which is slightly higher than a previous report of 7.1 years, but lower than the median age at death of 12.0 years reported across all dog breeds [4]. However, given the youthful and expanding population of Chihuahuas in the current study, the reported overall age at death should be interpreted cautiously to avoid confusion with breed-specific estimated lifespan. Given that the median age of 2.8 years for dogs that were alive in our study in 2016 is well below the median ages of dogs in breed-specific longevity studies, it might be that the bulk of our Chihuahua population were young during the study and therefore there were relatively few older dogs available to die in the study population. This would have the effect of biasing the age at death results downwards. Cohort studies that follow breeds from birth to death can provide the most robust epidemiological results but are often limited by high losses to follow-up while the long follow-up times needed can also be problematic [7].

Longevity comparisons between permanent characteristics within dogs are likely to have higher reliability. The median age at death of females (10.2 years) was significantly greater than males (6.9 years). A female longevity advantage as a general trend has been reported for dogs previously [26] but this is not universal across all breeds [27, 28]. Awareness of a female longevity advantage could assist prospective owners considering acquiring a Chihuahua, especially where the longevity disparity is quite marked. However, given the youthful population within this study, the mortality data were not extensive for this breed and the sex-association reported here is likely to be weighted towards early-life deaths. Therefore, future studies exploring sex-related longevity effects as the current cohort ages would be valuable to take account of the life-long health of these dogs.

The most common grouped causes of death were heart disease (18.8%), lower respiratory tract disorder (16.3%) and traumatic injury (13.8%). Analysis of 74,556 dog deaths from US veterinary teaching hospitals reported Chihuahuas with the third highest relative proportion (0.185) of cardiovascular causes of death out of the 82 breeds studied [29]. Chihuahuas have reported predisposition to pulmonic stenosis and patent ductus arteriosus (congenital heart conditions) and degenerative mitral valve disease (an acquired heart condition), which all carry a poor prognosis [30–33]. Taken together, heart disease is highlighted as an important life-limiting disorder in the Chihuahua that veterinarians should prioritise during routine examinations to facilitate early diagnosis and intervention. It is worth noting that the mortality data in the current study were relatively small and therefore future studies with greater numbers would offer more precise results.

Aggression was the fifth most common fine-level disorder in the current study and was recorded in 4.2% of Chihuahuas. This prevalence is higher than similarly-designed studies of other small-sized breeds; aggression did not feature among the most common fine-level disorders in Pugs [34], Border Terriers [35] or Miniature Schnauzers [13] and was the 13th most common disorder in French Bulldogs (2.3%) [28]. The Kennel Club describes the Chihuahua as “bold and saucy in temperament” and with “a huge personality in a tiny frame” [1]. These desired personality traits may predispose the Chihuahua towards aggressive tendencies. In a study spanning many breeds, those with the greatest percentage of dogs exhibiting serious aggression (bites or bite attempts) toward human strangers and owners included Dachshunds, Chihuahuas and Jack Russell Terriers [36]. Given that the Chihuahua has been reported as among the 10 dog breeds most commonly surrendered to animal shelters, often with undesirable behaviour cited as a contributing factor [37], the results of the current study further support aggression as an important issue in Chihuahuas. A study of veterinarians’ opinions regarding aggression in different dog breeds classified the Chihuahua as ‘very aggressive’ [38]. The Chihuahua has been reported as one of the most common breeds to exhibit ‘serious aggression’ (bites or bite attempts) toward humans (both strangers and owners), alongside other small breeds including the Dachshund and Jack Russell [36]. The small physical size of the Chihuahua may give buyers the perception that these dogs are easy to keep but Chihuahuas are generally full of energy, strong-willed and need exercise and mental stimulation just like any other dog [37]. Whether body size plays a part in either the prevalence of aggressive behavior in dogs, human perception of behavior as aggressive or human response to aggressive behaviors has not, to our knowledge, been reported yet. Aggressive behaviors preceding bites or bite attempts in the canine ‘ladder of aggression’ (e.g. stiffening up, staring, growling) may be more likely to be ignored or not taken seriously in miniature breeds such as the Chihuahua, leading to escalation to more serious behaviors including snaps and bites [39].

Aggression is a complex topic and can be highly context-dependent in dogs [40] and therefore the causes of aggression and resultant treatment plans are likely to vary widely between individual dogs. The current study identified a higher prevalence for aggression in males compared with females (4.9% versus 3.5%). A male predisposition to aggression is supported by a substantial body of evidence for several breeds [40–45]. A deeper understanding of this predisposition, including whether different interventions may be required for male or female dogs to either prevent the development of aggressive behavior, or treat it once established requires further investigation. As Chihuahuas increase in popularity, it is critically important that the quality of breeders remains high in order to produce puppies that show both physical and behavioural health. In a recent study, Chihuahuas acquired from less-responsible breeders were reported to show more aggression toward familiar dogs, unfamiliar dogs, unfamiliar humans and their owners than those acquired from breeders considered more responsible based on a number of husbandry factors [19]. As such, promoting improved breeding practices while making potential puppy buyers aware of the broad negative implications of purchasing puppies from less responsible breeders (and how to identify such breeders) is of great importance.

Periodontal disease was the most prevalent fine-level disorder recorded in the current study, affecting 13.5% of Chihuahuas, while dental disease overall affected 19.0% of the dogs in the study. This value is slightly lower than previous reports in other small-sized breeds such as the Cavalier King Charles Spaniel (15.2%) [46], Miniature Schnauzer (17.4%) [13] and Border Terrier (17.6%) [35]. However, the relatively youthful median age, small body size and relatively high comorbidity of retained deciduous dentition of the Chihuahuas in the current study needs to be carefully considered when interpreting these findings. Periodontal disease prevalence increases with age, increases with decreasing body size [47] and increases with dental malocclusion [48]**.** Given that the median age of Chihuahuas in the current study was 2.8 years, the median adult bodyweight overall was 3.4 kg and that 5.7% of dogs were recorded with retained deciduous dentition, the current results do suggest that the breed should be considered as predisposed to dental disease. This conclusion is reinforced by the finding in the current study that retained deciduous dentition was the third most prevalent fine-level disorder. Purebred toy breeds are reported in general with a predisposition to retained deciduous dentition that can result in dental malocclusion and promotion of acquired dental problems [49]. The Kennel Club cites incorrect dentition in the Chihuahua as a point of concern for special attention by judges [9].

Obesity is increasingly being recognised as a disease with significant health and welfare consequences for affected dogs [50]. Obesity was the second most prevalent fine-level disorder diagnosed in Chihuahuas, affecting 5.9% of the study population. Whilst the prevalence of obesity in Chihuahuas is lower than the prevalence reported in other breeds such as the Border Terrier (7.0%) [35], Miniature Schnauzer (8.3%) [13] and Pug (13.2%) [34], the high rank of obesity among the commonly recorded conditions still marks this condition out as important for the breed. Given that increasing age is a risk factor for obesity, with dogs aged 5 to 10 years particularly prone, the youthful population of Chihuahuas in the current study suggests that this prevalence will increase as this cohort ages and therefore further emphasises the relevance of weight management in this breed [51].

The Kennel Club breed standard for the Chihuahua specifies a bodyweight up to 2.7 kg, with 1.8–2.7 kg preferred [1]. However, the median adult bodyweight of Chihuahuas in the current study that represent a mix of pedigreed and non-pedigreed dogs was substantially larger at 3.4 kg. Without a concomitant body condition score assessment, it is difficult to determine the contribution, if any, of obesity on the current bodyweight of individual dogs [52]. However, the substantial difference between the specified bodyweights for pedigreed dogs and the actual bodyweights of the wider population of Chihuahuas in the UK suggests that the general public prefer a larger type of this breed that is heavier than the breed standard. The contrast between the breed standards and the reality of bodysize in the general population of Chihuahuas in the UK reported here suggests that the bodyweight limits specified in the Kennel Club breed standard should be further explored [53]..

The results of the current study support previous reports that the Chihuahua is predisposed to patellar luxation and cryptorchidism [54–57]. Patellar luxation affected 4.0% of Chihuahuas and cryptorchidism affected 3.9% of male Chihuahuas in the current study. Patellar luxation, which has been reported to be one of the five most important hereditary defects in dogs from a welfare impact perspective [58, 59], is more common in smaller breed dogs [56]. Smaller breed dogs, such as the Miniature Poodle, Pomeranian and Yorkshire Terrier, have also been identified as at risk of cryptorchidism which is also considered as an inheritable disorder [57, 60]. Some dogs can show both of these disorders concurrently and therefore a link between the disorders has been postulated [57]. Increasing risk with reducing physical size of dogs to patellar luxation, cryptorchidism and retained deciduous dentition could suggest benefits from breeding away from the diminutive extreme conformations of the Chihuahua to improve the overall health and welfare of the breed [47, 60–62].

The study had an a priori interest in exploring sex-related differences. As discussed above, males had reduced age at death and increased prevalence of aggression compared to females. In addition, the current results suggest that male Chihuahuas may have poorer health in general than female Chihuahuas. The median annual disorder count was statistically higher (Mann-Whitney U test: *P* <  0.001) in males than in females. Among the 28 most common fine-level disorders, males had statistically (chi-square test, *P* <  0.05) higher probability than females for 5 disorders (aggression, heart murmur, otitis externa, conjunctivitis and upper respiratory tract infection), whilst there were no disorders with statistically (chi-square test, *P* <  0.05) higher prevalence in females. Whilst the underlying cause of the heart murmurs recorded in the current study is unknown, Chihuahuas are reportedly predisposed to degenerative mitral valve disease and pulmonic stenosis [30, 31, 33] which have both been reported as more common in male dogs in recent studies [30, 31], although an older study reported a greater risk in females [33]. There is conflicting previous evidence regarding sex predisposition to otitis externa. Although several studies have failed to show sex-related differences in otitis externa [27, 28, 35, 43, 44, 63], a report based on 273 dogs presenting to teaching and referral hospitals in India identified a higher prevalence of otitis externa in male dogs compared to females [64]. Androgenic hormones may increase sebum production, which is a predisposing factor to flare up of latent otic infection, whereas oestrogens elicit an opposite response [64]. A significantly increased prevalence of conjunctivitis and upper respiratory tract infection in male dogs has also been reported in the French bulldog [28]. Both the Chihuahua and French bulldog are popular breeds with extreme physical features [18]. The discovery and reporting of sex-based prevalence differences highlights that certain disorders may benefit from specific focus within sexes in order to contribute to improved health and welfare.

Male Chihuahuas were significantly more likely to be neutered than female Chihuahuas (31.8% versus 22.0%). This contrasts to the findings of many other breed-related studies that reported higher proportional neutering in females [13, 27, 34, 35, 43, 44] although male French Bulldogs were also more likely to be neutered than females [28]. Both Chihuahuas and French Bulldogs are breeds with rapidly rising popularity and consequently youthful populations. It is possible that owners are more willing to neuter male dogs of these breeds at an earlier age whereas thoughts about using the bitches as breeding stock may deter early neutering of females. Additionally, a real or perceived tendency to aggression in male Chihuahuas may also encourage owners to preferentially request neutering of male animals. Several studies have reported that entire male dogs are disproportionately more likely to display aggressive behavior compared to neutered dogs [45, 65, 66].

There are limitations to the application of veterinary clinical records for epidemiological research that have been reported previously [67, 68]. In addition to these, it is worth noting that a final specified biomedical diagnosis is not always reached, or potentially even required, for effective clinical management in the primary-care setting [69, 70]. A subset of dogs in the overall UK dog population are unregistered with a veterinary practice. If the prevalence of, and risk factors for, disease in this unregistered group differs to the subset that are under veterinary care, then the results of the current study may not generalise well to this wider and unrecorded group. As discussed, the median age of Chihuahuas in the current study was 2.8 years and therefore the current results may be skewed towards disorders of younger dogs. The need to consider impacts from the median age of the population when interpreting the results of any study is emphasised by the statistically significant (chi-square or Fisher’s exact test, *P* <  0.05) differences in prevalence between the three age bands identified in 73.3% of the common disorders assessed. The current study made no distinction between smooth and long coat varieties of Chihuahua. Lowering counts of bodyweight data points in Fig. 2 as the dogs aged reduced the certainty of interference for older ages.

## Conclusion

This study of over eleven thousand Chihuahuas under primary veterinary care documented rising ownership of Chihuahuas in the UK. This popularity boom suggests that veterinarians need to be watchful for welfare issues related to impulse purchase of Chihuahua puppies by owners with limited experience of dog care. This youthful population may have generated results that over-represent the currently young demographic of this breed in the UK. Heart disease was the most common cause of death. The most prevalent disorders were periodontal disease, obesity, retained deciduous dentition, anal sac impaction and aggression. Given the youthful population, the prevalence of some conditions such as periodontal disease and obesity are only likely to increase with age. Some important sex-associated differences were identified, with males showing earlier age at death and having higher prevalence of aggression, heart murmur, otitis externa, conjunctivitis and upper respiratory tract infection. The unique veterinary care needs of this popular miniature breed suggest that veterinarians should consider the value of advanced training in anesthesia and dentistry in small-sized dogs.

## Methods

The denominator population for the study covered all dogs within VetCompass in 2016 that were under primary veterinary care. The criteria for being ‘under veterinary care’ required a) ≥ 1 electronic patient record (EPR) during 2016 (bodyweight, free-text clinical note, treatment, VeNom diagnosis term) and/or b) ≥ 1 EPR during both 2015 and 2017. VetCompass is a research programme that shares anonymized clinical records from primary-care veterinary practices in the UK [14]. These shared data fields include a unique animal ID linked to species, breed, sex, neuter, date of birth, colour, bodyweight, along with free-form text clinical notes, summary diagnosis terms [71] and treatment with associated dates. It is noteworthy that the design and analysis of the current study deliberately parallelled those used in some previous VetCompass breed-based studies in order to facilitate reliable comparisons between dog breeds [13, 44, 72, 73].

A *2016 period cross-sectional* study design was used to estimate the one-year (2016) period prevalence of the most commonly diagnosed disorders [74]. Power calculations showed that 4452 dogs were needed to estimate the prevalence of a disorder that occurred in 3% of dogs with 0.5% acceptable margin of error at a 95% confidence level [75]. Ethics approval was obtained from the RVC Ethics and Welfare Committee (reference number 2015/1369). Owners of all dogs included in VetCompass consented to share anonymized clinical data relating to these dogs.

Dogs recorded as Chihuahua breed were categorised as Chihuahua and all remaining dogs were categorised as non-Chihuahua. No distinction was made between Kennel Club registered and unregistered individuals or between smooth and long coat varieties. *Adult Bodyweight* showed the mean bodyweight (kg) recorded from all bodyweight information for dogs over 18 months at time of weighing and was grouped as < 2.0, 2.0 to < 3.0, 3.0 to < 4.0, 4.0 to < 5.0, 5.0 to < 6.0 and ≥ 6.0. *Neuter* described the recorded status (entire or neutered) at the final EPR. *Age* showed the age in years at December 31st, 2016 and was grouped as < 1.0, 1.0 to < 2.0, 2.0 to < 3.0, 3.0 to < 5.0, 5.0 to < 7.0, 7.0 to < 9.0, 9.0 to < 11.0 and ≥ 11.0.

Clinical records were manually reviewed from a random subset of dogs and the most precise diagnosis terms for all disorders that existed during 2016 were extracted [15]. Non-therapeutic clinical interventions were excluded. Incident and pre-existing presentations for disorders were not differentiated. Events that were not recorded with a standard diagnostic term were coded to the first presenting clinical sign stated in the clinical notes. Information on the random sample of dogs was extracted for all deaths at any available date to describe the cause, date and method of death The full lists of diagnosis terms were mapped to both fine-level precision and grouped-level precision hierarchies of diagnostic precision as described previously [15]. Fine-level precision terms provided disorder information to maximal high level of diagnostic precision available within the clinical notes (e.g. *nodular episcleritis* would map to *episcleritis*) while grouped-level precision terms provided information to a more general level of diagnostic precision (e.g. *nodular episcleritis* would map to *ophthalmological disorder*).

Data checking and cleaning used Excel (Microsoft Office Excel 2013, Microsoft Corp.) and analysis used Stata Version 13 (Stata Corporation). Results for Chihuahuas under veterinary care in 2016 were reported for sex, neuter status, age, colour and adult bodyweight. Annual proportional birth rates described proportional counts of Chihuahuas born annually from 2005 to 2016 compared with all dogs that were under veterinary care in 2016. All available bodyweight and age data contributed to individual bodyweight growth curves for male and female Chihuahuas. Age-specific bodyweights were plotted and overlaid with a cross medians line plot (Stata *mband* command).

The one-year period prevalence values described the probability of at least one diagnosis of that disorder during 2016. The 95% confidence intervals (CI) estimates were calculated from standard errors based on approximation to the binomial distribution [76]. The median age was shown for the final study date for affected animals. Prevalence values were reported overall and also separately for males and females. The chi-square test was used to compare categorical variables (Fisher’s exact test was used if at least one of the reported cells was under 5) and the Students t-test or Mann-Whitney U test to compare continuous variables as appropriate [76]. Statistical significance was set at the 5% level.

## Data Availability

The datasets generated and/or analysed during the current study are available in the RVC repository, http://researchonline.rvc.ac.uk/12182/

## Abbreviations

CI: Confidence interval
EPR: Electronic patient record
IQR: Interquartile range
OR: Odds ratio
SD: Standard deviation
